# Disruption of the potassium channel regulatory subunit *KCNE2* causes iron-deficient anemia

**DOI:** 10.1016/j.exphem.2014.07.269

**Published:** 2014-12

**Authors:** Grace Salsbury, Emma L. Cambridge, Zoe McIntyre, Mark J. Arends, Natasha A. Karp, Christopher Isherwood, Carl Shannon, Yvette Hooks, The Sanger Mouse Genetics Project, Ramiro Ramirez-Solis, David J. Adams, Jacqueline K. White, Anneliese O. Speak

**Affiliations:** a Wellcome Trust Sanger Institute, Wellcome Trust Genome Campus, Hinxton, Cambridgeshire, United Kingdom; b University of Edinburgh Division of Pathology, Institute of Genetics & Molecular Medicine, Western General Hospital, Edinburgh, United Kingdom

## Abstract

Iron homeostasis is a dynamic process that is tightly controlled to balance iron uptake, storage, and export. Reduction of dietary iron from the ferric to the ferrous form is required for uptake by solute carrier family 11 (proton-coupled divalent metal ion transporters), member 2 (Slc11a2) into the enterocytes. Both processes are proton dependent and have led to the suggestion of the importance of acidic gastric pH for the absorption of dietary iron. Potassium voltage-gated channel subfamily E, member 2 (KCNE2), in combination with potassium voltage-gated channel, KQT-like subfamily, member 1 (KCNQ1), form a gastric potassium channel essential for gastric acidification. Deficiency of either *Kcne2* or *Kcnq1* results in achlorhydia, gastric hyperplasia, and neoplasia, but the impact on iron absorption has not, to our knowledge, been investigated. Here we report that *Kcne2*-deficient mice, in addition to the previously reported phenotypes, also present with iron-deficient anemia. Interestingly, impaired function of KCNQ1 results in iron-deficient anemia in Jervell and Lange-Nielsen syndrome patients. We speculate that impaired function of KCNE2 could result in the same clinical phenotype.

Iron is imperative for human health, and defects in iron homeostasis are known to result in serious pathologic abnormalities such as hemochromatosis and anemia. This dynamic process requires a constant balance of iron achieved by both intake of dietary iron and successful coordination of iron uptake, export, and storage. Iron-deficient anemia can be caused by a lack of dietary iron, blood loss, or a physiologic defect affecting iron bioavailability, uptake, or transfer into the circulation. The majority of dietary iron is in the ferric form and requires reduction to the ferrous form before being transported by solute carrier family 11 (proton-coupled divalent metal ion transporters), member 2 (Slc11a2), which is located in the brush border of the enterocytes [1].

Potassium voltage-gated channel subfamily E, member 2 (KCNE2) is a single-pass integral membrane β-subunit of a potassium ion channel and assembles with various α-subunits. In a heterotrimeric channel with potassium voltage-gated channel, KQT-like subfamily, member 1 (KCNQ1), KCNE2 forms a constitutive potassium ion channel at the apical membrane of gastric parietal cells [2]. This KCNE2/KCNQ1 potassium channel provides a constant source of potassium ions into the stomach lumen. The ions are used by the gastric K^+^/H^+^-ATPase to pump hydrogen ions into the stomach lumen [3]. Point mutations in *KCNE2* have been shown to cause Long QT Syndrome 6 [4], a phenotype recapitulated in knockout mouse models of *Kcne2* [5]. In addition, *Kcne2*-deficient mice have been reported to have gastric hyperplasia and neoplasia, achlorhydria 3, 6, anemia [7], and hypothyroidism [8]. Gastric pH has been suggested to be a critical determinant for dietary iron absorption, a theory supported by the observation that the sublytic mouse model, with a point mutation in *Atp4a* (K^+^/H^+^-ATPase α-subunit), has increased gastric pH and iron-deficient anemia [9].

In this study, we have generated a targeted gene trap for *Kcne2* and identified that mutant male animals suffer from iron-deficient anemia.

## Materials and methods

### Animals

Generation of the *Kcne2*^*tm1a(EUCOMM)Wtsi*^ allele (hereafter referred to as *Kcne2*^*tm1a*^) was performed as part of the European Conditional Mouse Mutagenesis Program and Knockout Mouse Project (EUCOMM/KOMP) projects and Sanger Mouse Genetics Project [10]. Mice were generated from embryonic stem cell clone EPD0156_2_F10 and backcrossed to C57BL/6N females, with genotyping carried out as previously described [11]. Animals were housed in specific pathogen-free conditions and placed on a Western high fat diet (Special Diet Services, Witham, UK) from 4 weeks of age with ad libitum access to autoclaved, nonacidified water and food and phenotyped according to a standard pipeline, as previously reported [12]. All experiments were performed in accordance with the UK Home Office regulations, UK Animals (Scientific Procedures) Act 1986.

### Blood sample collection

At 16 weeks, blood was collected by puncture of the retro-orbital sinus under terminal anaesthesia within 1−3 hours of lights on and collected into ethylenediaminetetraacetic acid-coated tubes (Kabe Labortechnik, Numbrecht, Germany) for hematology (Scil Vetabc, Montpellier, France) and into heparinized tubes (Kabe Labortechnik) for plasma preparation. A total of 26 parameters were determined from plasma using an Olympus AU400 analyzer (Beckman Coulter, High Wycombe, UK). Insulin and erythropoietin were determined using a Meso Scale Discovery array (Rockville, MD) and interleukin 6 was measured by enzyme-linked immunosorbent assay (eBioscience, Hatfield, UK).

### Histopathology

Full necropsy was performed on two male and two female *Kcne2*^*tm1a/tm1a*^ mice and two controls of each sex. All tissues were collected, fixed in formalin, and embedded in paraffin wax according to standard protocols. Sections were cut and stained with haematoxylin and eosin or Perls' Prussian blue according to standard methods.

### Data analysis and statistics

For all data except transferrin, ferritin, and erythropoietin, the impact of genotype was assessed using a mixed-model framework as described [13]. For each phenotypic trait tested, the global *p* value was adjusted to account for multiple comparisons to control the false discovery rate to 5% (*R* function: *p* = 0.0163), and the adjusted value is reported in the text. The genotype *p* value is indicated on the figures, and the full details are listed in Table 1. Transferrin, ferritin, and erythropoietin were analyzed using a one-way ANOVA using Sidak's multiple comparisons test and adjusting for multiple testing using Prism v6 (GraphPad, San Diego, CA).

**Table 1:** Mixed-model output for the significant hematology and plasma chemistry parameters

Variable	Global test	Sexual dimorphism	Genotype effect		Genotype*Female		Genotype*Male		Classification
	*p* value	*p* value	Effect size	*p* value	Effect size	*p* value	Effect size	*p* value	
Red blood cell count	4.35 × 10^−4^	1.00 × 10^−4^			0.523 × 10^6^	0.157	−1.30 × 10^6^	3.87 × 10^−3^	Males only
Hemoglobin	3.60 × 10^−4^	2.00 × 10^−4^			0.305	0.684	−3.635	4.38 × 10^−5^	Males only
Hematocrit	7.30 × 10^−4^	4.00 × 10^−4^			0.482	0.826	−10.117	1.00 × 10^−4^	Males only
Mean corpuscular hemoglobin	2.96 × 10^−3^	0.0426			−0.510	0.273	−1.840	5.00 × 10^−4^	Males only
Red blood cell distribution width	0	1.37 × 10^−8^			0.174	0.225	1.345	3.66 × 10^−19^	Males only
Mean corpuscular volume	7.50 × 10^−4^	0.0413			−1.713	0.136	−5.033	2.20 × 10^−5^	Males only
Platelet count	9.90 × 10^−4^	5.20 × 10^−3^			66250	0.579	5.392 × 10^5^	6.44 × 10^−5^	Males only
Iron	1.09 × 10^−8^	0.236	−14.244	2.71 × 10^−7^					Both sexes equally
Magnesium	1.62 × 10^−5^	0.532	0.1257	1.00 × 10^−4^					Both sexes equally

Parameters were assessed by a significance threshold of <0.0163 on the global test output. This threshold was selected to manage multiple testing and control the false discovery rate to 5%. The global test *p* value is a test of the genotype impact. The methodology assesses for sexual dimorphism, and, when significant (sexual dimorphism *p* value <0.05), the model will estimate the genotype effect for each sex separately (Genotype*Female and Genotype*Male); based on the significance of the *p* values for each sex effect, the genotype effect can be classified (e.g., male only). When sexual dimorphism was not significant, the data from both sexes were combined to assess the overall genotype effect (Genotype Effect).

## Results and discussion

**Figure 1:**
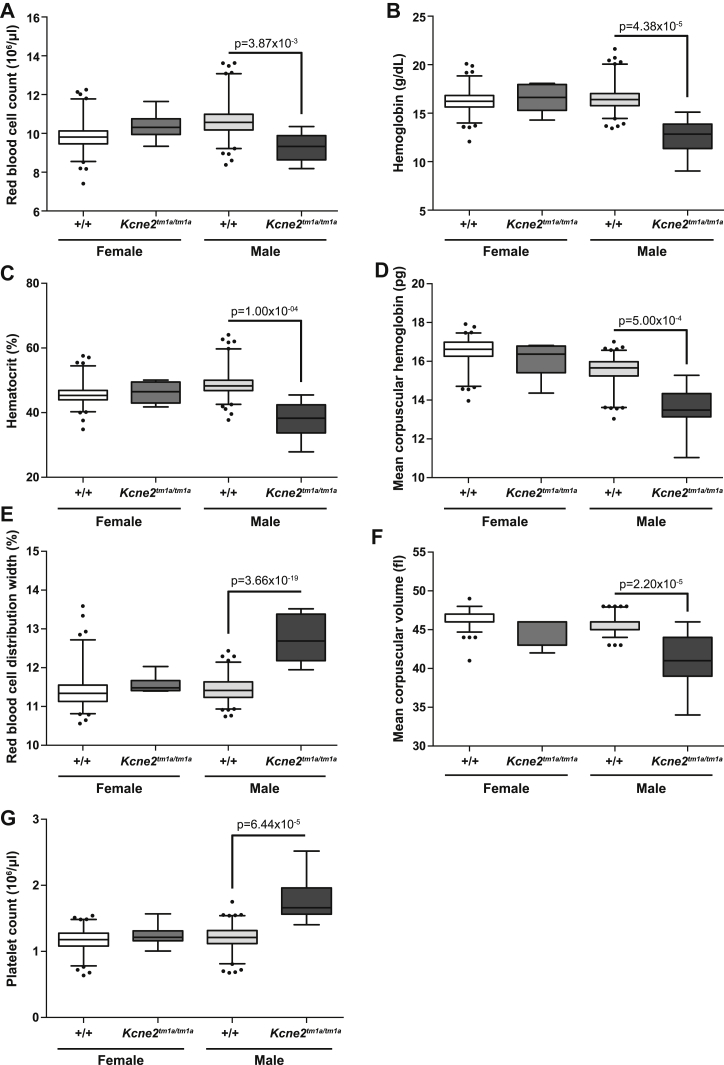
Altered hematologic parameters in *Kcne2*^*tm1a/tm1a*^ mutants. (**A**) Red blood cell count, (**B**) hemoglobin, (**C**) hematocrit, (**D**) mean corpuscular hemoglobin, (**E**) red blood cell distribution width, (**F**) mean corpuscular volume, and (**G**) platelet count were all determined at 16 weeks of age. For male control versus male *Kcne2*^*tm1a/tm1a*^ mice, *p* values are indicated with the boxplots showing the mean interquartile range, with whiskers to the 2.5 and 97.5 percentiles and dots for outliers. For all graphs, *n* = 7 for female *Kcne2*^*tm1a/tm1a*^ mutants, *n* = 187 for female controls, *n* = 7 for male *Kcne2*^*tm1a/tm1a*^ mutants, and *n* = 202 for male controls.

Seven hematologic parameters were significantly different in male *Kcne2*^*tm1a/tm1a*^ mutants compared with controls (Table 1; Supplementary Table E1, online only, available at www.exphem.org). There was a decrease in the red blood cell count (*p* = 4.35 × 10^−4^; Fig. 1A), hemoglobin (*p* = 3.60 × 10^−4^; Fig. 1B), hematocrit (*p* = 7.30 × 10^−4^; Fig. 1C) and mean corpuscular hemoglobin (*p* = 2.96 × 10^−3^; Fig. 1D). This was accompanied by increased red blood cell distribution width (*p* = 0; Fig. 1E) and decreased mean corpuscular volume (*p* = 7.50 × 10^−4^; Fig. 1F). These altered red blood cell indices are indicative of hypochromic microcytic anemia and are in agreement with a recent report [7]. There was also evidence of reactive thrombocytosis, with an increased platelet count in the male *Kcne2*^*tm1a/tm1a*^ mutants (*p* = 9.90 × 10^−4^; Fig. 1G). Interestingly, no significant hematologic differences were detected in females.

**Figure 2:**
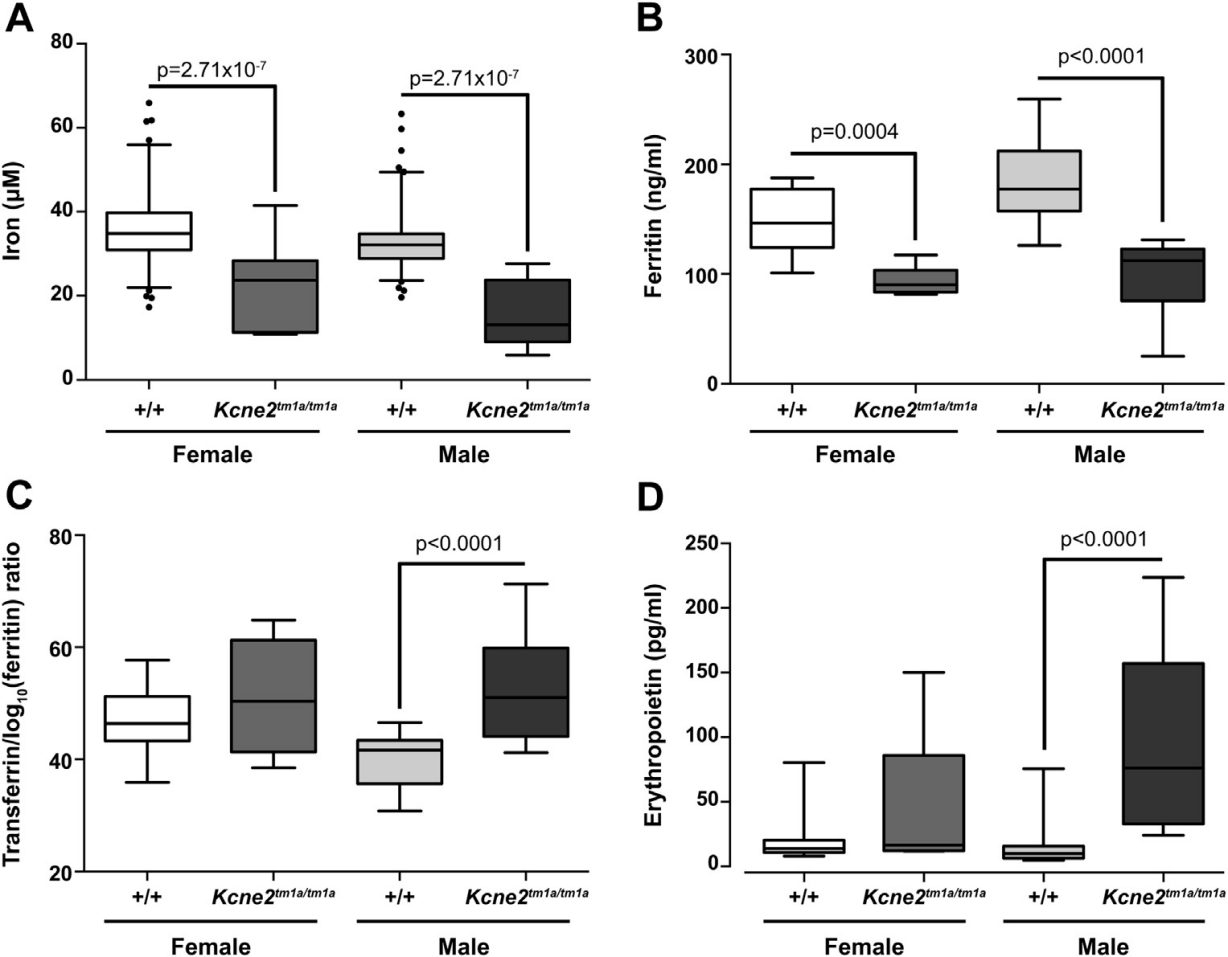

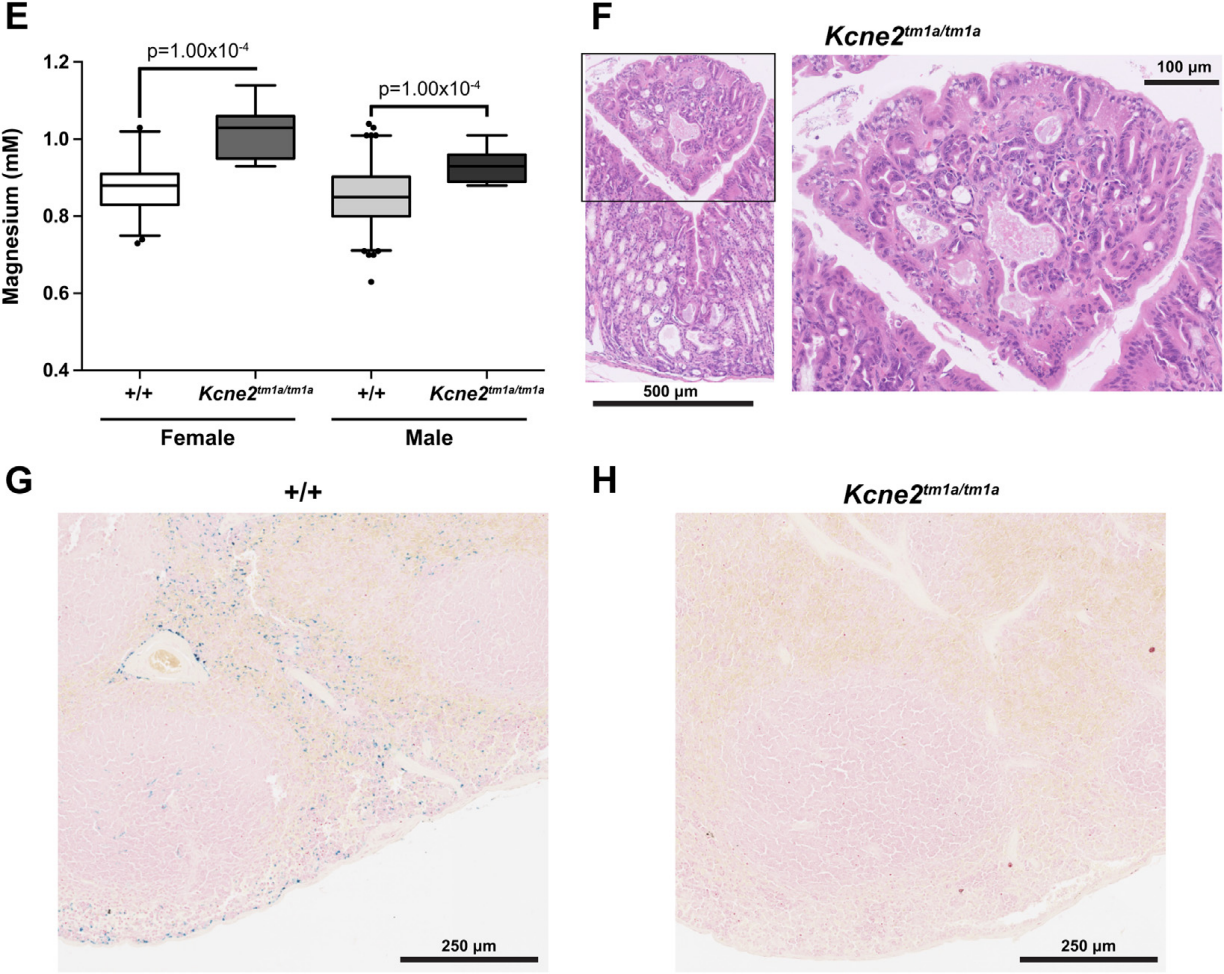
Altered plasma chemistry parameters in *Kcne2*^*tm1a/tm1a*^ mutants. (**A**) Iron, (**B**) ferritin, (**C**) transferrin/log_10_(ferritin) ratio, (**D**) erythropoietin, and (**E**) magnesium were all determined at 16 weeks of age. For the genotype effect of male control versus male *Kcne2*^*tm1a/tm1a*^ mice, *p* values are indicated with boxplots showing the mean interquartile range, with whiskers to the 2.5 and 97.5 percentiles and dots for outliers. For iron, magnesium, ferritin, and transferrin, *n* = 7 for *Kcne2*^*tm1a/tm1a*^ female and male mutants; for erythropoietin, *n* = 5 for female and *n* = 6 for male *Kcne2*^*tm1a/tm1a*^ mutants. For iron and magnesium, *n* = 186 female and *n* = 202 male controls. For erythropoietin, *n* = 19 female and *n* = 21 male controls; for ferritin, *n* = 21 female and *n* = 23 male controls; for transferrin, *n* = 22 for female and *n* = 23 for male controls. (**F**) Presence of a gastric adenoma (surrounded by the box), with architectural and nuclear atypia typical of a dysplastic adenoma, in a male *Kcne2*^*tm1a/tm1a*^ mutant. In male *Kcne2*^*tm1a/tm1a*^ mutants, we observed reduced iron content in spleen, as detected by Perls' Prussian blue stain; shown are representative images from (**G**) a male control and (**H**) a male *Kcne2*^*tm1a/tm1a*^ mutant.

We analyzed in detail the plasma chemistry parameters with a focus on those that could correlate with anemia (Table 1; Supplementary Table E1, online only, available at www.exphem.org). There was a significant decrease in the plasma iron concentration in both male and female *Kcne2*^*tm1a/tm1a*^ mutants compared with the controls (*p* = 1.09 × 10^−8^; Fig. 2A), suggestive of iron-deficient anemia. It has previously been demonstrated that Kcne2 is essential for gastric acid secretion and that *Kcne2*-deficient mice have an increased stomach pH [3]. Because it has also been demonstrated that a low gastric pH is required for absorption of dietary iron [9], we hypothesize that the increased gastric pH in *Kcne2*^*tm1a/tm1a*^ mutants could account for the low plasma iron and iron-deficient anemia. To support this finding, we tested plasma ferritin, transferrin, and erythropoietin. We observed a significant decrease in the plasma ferritin concentration in both male and female *Kcne2*^*tm1a/tm1a*^ mutants compared with the controls (*p* < 0.0001; Fig. 2B). There was a trend to increased transferrin, although this was not significant. However, using the transferrin/log_10_(ferritin) ratio, suggested to be a sensitive indicator of iron-deficient anemia [14], there was a significant increase in male *Kcne2*^*tm1a/tm1a*^ mutants compared with the controls (*p* < 0.0001; Fig. 2C). Erythropoietin was significantly increased in male *Kcne2*^*tm1a/tm1a*^ mutants compared with controls (*p* < 0.0001; Fig. 2D). Erythropoietin and transferrin/log_10_(ferritin) ratios were only significantly different in the males, which could account, in part, for the observation of hematologic abnormalities only in males. We hypothesize that this finding could be linked to the differential effects of sex hormones on regulating iron stores and erythropoiesis. Plasma magnesium was significantly increased in both male and female *Kcne2*^*tm1a/tm1a*^ mutants compared with controls (*p* = 1.62 × 10^−5^; Fig. 2E), although the significance of this finding is unclear. In contrast to the result observed by Hu et al. [7], there was no significant difference in potassium and no evidence of dyslipidemia or altered glucose tolerance.

To investigate other causal factors, we performed a full histologic assessment, and, in agreement with previous reports 3, 6, *Kcne2*^*tm1a/tm1a*^ mutant mice display gastric hyperplasia, abnormal parietal cell morphology, and decreased numbers of chief cells. Inflammation and neutrophil infiltration in the gastric mucosa were also observed in *Kcne2*^*tm1a/tm1a*^ mutants. The abnormalities were more severe in the two male samples as compared with the females and could be linked to a more extreme response among the males to high fat diet challenge. Such a diet challenge has previously been demonstrated to have a heightened inflammatory response in males [15]. One of the male *Kcne2*^*tm1a/tm1a*^ mutants presented with a gastric adenoma (Fig. 2F, box) previously observed in aged *Kcne2*-deficient mice [6] and *Kcnq1* mutants [16]. There was no indication of disruptions to the small intestine villi, and the bone marrow and spleen did not exhibit any gross abnormalities between *Kcne2*^*tm1a/tm1a*^ mutants and controls. The livers of both controls and *Kcne2*^*tm1a/tm1a*^ mutants exhibited indications of nonalcoholic fatty liver disease, consistent with being placed on a high fat diet for 12 weeks [17]. Upon staining with Perls' Prussian blue to assess iron stores, distinct blue staining could be detected in the spleen sections from controls (Fig. 2G), but this was virtually undetectable in all four *Kcne2*^*tm1a/tm1a*^ samples (Fig. 2H).

The link between inflammation, particularly proinflammatory cytokines, and alterations to iron homeostasis is well established [18]. Since we observed inflammation in our histologic examination of *Kcne2*^*tm1a/tm1a*^ mutants, we determined the concentration of cytokines in the plasma. We found that interleukin 6 levels were below 50 pg/mL in all *Kcne2*^*tm1a/tm1a*^ mutants and controls. This further strengthens the view that the hematologic abnormalities observed are due to iron deficiency and are not the result of systemic inflammation.

In conclusion, this study has provided further evidence for the importance of gastric pH-regulating mechanisms in the absorption of dietary iron, the malfunction of which can lead to the development of iron-deficient anemia. Both sexes presented with decreased plasma iron, whereas only the males developed anemia; we speculate that this effect is linked to the differential effect of sex hormones on iron stores and erythropoiesis [19]. Interestingly, these findings could be clinically relevant, as it was recently reported that impaired function of KCNQ1 in Jervell and Lange-Nielsen syndrome results in iron-deficient anemia and gastric hyperplasia [15]. Given the similarities in the gastric phenotype of *Kcne2*- and *Kcnq1*-deficient mice, we speculate that impaired function of KCNE2 could result in a similar clinical presentation.

## Figures and Tables

**Supplementary Table E1:** Full breakdown of hematology and plasma chemistry parameters showing *n*, mean, and standard deviation

Variable	Female				Male			
	+/+		*Kcne2*^*tm1a/tm1a*^		+/+		*Kcne2*^*tm1a/tm1a*^	
	*n*	Mean ± SD	*n*	Mean ± SD	*n*	Mean ± SD	*n*	Mean ± SD
Red blood cell count (×10^6^/μL)	187	9.87 ± 0.68	7	10.35 ± 0.72	202	10.69 ± 0.86	7	9.28 ± 0.74
Hemoglobin (g/dL)	187	16.2 ± 1.13	7	16.5 ± 1.40	202	16.5 ± 1.25	7	12.7 ± 1.93
Hematocrit (%)	187	45.7 ± 3.10	7	46.1 ± 3.18	202	48.9 ± 3.94	7	37.9 ± 5.77
Mean corpuscular hemoglobin (pg)	187	16.5 ± 0.74	7	16.0 ± 0.90	202	15.5 ± 0.76	7	13.5 ± 1.30
Red blood cell distribution width (%)	187	11.4 ± 0.42	7	11.6 ± 0.22	202	11.5 ± 0.31	7	12.8 ± 0.59
Mean corpuscular volume (fl)	187	46.3 ± 0.92	7	44.6 ± 1.81	202	45.8 ± 0.94	7	40.7 ± 3.82
Platelet count (×10^6^/μL)	187	1.18 ± 0.16	7	1.24 ± 0.17	202	1.21 ± 0.17	7	1.78 ± 0.37
Iron (μmol/L)	186	35.8 ± 7.7	7	23.7 ± 10.5	202	32.6 ± 5.9	7	15.4 ± 7.9
Ferritin (ng/mL)	21	149.8 ± 28.3	7	93.4 ± 12.9	23	149.8 ± 28.3	7	95.8 ± 37.4
Transferrin (mg/dL)	22	103.1 ± 13.1	7	101.6 ± 17.7	23	90.2 ± 12.69	7	99.8 ± 6.7
Erythropoietin (pg/mL)	19	20.1 ± 17.8	5	42.6 ± 60.3	21	14.2 ± 15.4	6	95.2 ± 78.1
Magnesium (mmol/L)	186	0.88 ± 0.06	7	1.02 ± 0.07	202	0.85 ± 0.07	7	0.94 ± 0.05

## References

[1] Gunshin H., Fujiwara Y., Custodio A.O., Direnzo C., Robine S., Andrews N.C. (2005). Slc11a2 is required for intestinal iron absorption and erythropoiesis but dispensable in placenta and liver. J Clin Invest.

[2] Heitzmann D., Grahammer F., von Hahn T. (2004). Heteromeric KCNE2/KCNQ1 potassium channels in the luminal membrane of gastric parietal cells. J Physiol.

[3] Roepke T.K., Anantharam A., Kirchhoff P. (2006). The KCNE2 potassium channel ancillary subunit is essential for gastric acid secretion. J Biol Chem.

[4] Isbrandt D., Friederich P., Solth A. (2002). Identification and functional characterization of a novel KCNE2 (MiRP1) mutation that alters HERG channel kinetics. J Mol Med (Berl).

[5] Roepke T.K., Kontogeorgis A., Ovanez C. (2008). Targeted deletion of kcne2 impairs ventricular repolarization via disruption of I(K,slow1) and I(to,f). FASEB J.

[6] Roepke T.K., Purtell K., King E.C., La Perle K.M., Lerner D.J., Abbott G.W. (2010). Targeted deletion of KCNE2 causes gastritis cystica profunda and gastric neoplasia. PLoS One.

[7] Hu Z., Kant R., Anand M. (2014). KCNE2 deletion creates a multisystem syndrome predisposing to sudden cardiac death. Circ Cardiovasc Genet.

[8] Roepke T.K., King E.C., Reyna-Neyra A. (2009). KCNE2 deletion uncovers its crucial role in thyroid hormone biosynthesis. Nat Med.

[9] Krieg L., Milstein O., Krebs P., Xia Y., Beutler B., Du X. (2011). Mutation of the gastric hydrogen-potassium ATPase alpha subunit causes iron-deficiency anemia in mice. Blood.

[10] Skarnes W.C., Rosen B., West A.P. (2011). A conditional knockout resource for the genome-wide study of mouse gene function. Nature.

[11] Ryder E., Gleeson D., Sethi D. (2013). Molecular characterization of mutant mouse strains generated from the EUCOMM/KOMP-CSD ES cell resource. Mamm Genome.

[12] White J.K., Gerdin A.K., Karp N.A. (2013). Genome-wide generation and systematic phenotyping of knockout mice reveals new roles for many genes. Cell.

[13] Karp N.A., Melvin D., Mott R.F. (2012). Robust and sensitive analysis of mouse knockout phenotypes. PLoS One.

[14] Castel R., Tax M.G., Droogendijk J. (2012). The transferrin/log(ferritin) ratio: a new tool for the diagnosis of iron deficiency anemia. Clin Chem Lab Med.

[15] Grove K.L., Fried S.K., Greenberg A.S., Xiao X.Q., Clegg D.J. (2010). A microarray analysis of sexual dimorphism of adipose tissues in high-fat-diet-induced obese mice. Int J Obes (Lond).

[16] Elso C.M., Lu X., Culiat C.T. (2004). Heightened susceptibility to chronic gastritis, hyperplasia and metaplasia in KCNQ1 mutant mice. Hum Mol Genet.

[17] Podrini C., Cambridge E.L., Lelliott C.J. (2013). High-fat feeding rapidly induces obesity and lipid derangements in C57BL/6N mice. Mamm Genome.

[18] Nemeth E., Rivera S., Gabayan V. (2004). IL-6 mediates hypoferremia of inflammation by inducing the synthesis of the iron regulatory hormone hepcidin. J Clin Invest.

[19] Murphy W.G. (2014). The sex difference in haemoglobin levels in adults - mechanisms, causes, and consequences. Blood Rev.

