# Corrigendum to “Disruption of the potassium channel regulatory subunit *KCNE2* causes iron-deficient anemia” [Experimental Hematology, Vol. 42, Issue 12, p1053–1058.e1]

**DOI:** 10.1016/j.exphem.2015.03.002

**Published:** 2015-05

**Authors:** Grace Salsbury, Emma L. Cambridge, Zoe McIntyre, Mark J. Arends, Natasha A. Karp, Christopher Isherwood, Carl Shannon, Yvette Hooks, The Sanger Mouse Genetics Project, Ramiro Ramirez-Solis, David J. Adams, Jacqueline K. White, Anneliese O. Speak

**Affiliations:** a Wellcome Trust Sanger Institute, Wellcome Trust Genome Campus, Hinxton, Cambridgeshire, United Kingdom; b University of Edinburgh Division of Pathology, Institute of Genetics & Molecular Medicine, Western General Hospital, Edinburgh, United Kingdom

The authors regret that an incorrect reference was cited in the discussion section. “Interestingly, these findings could be clinically relevant, as it was recently reported that impaired function of KCNQ1 in Jervell and Lange-Nielsen syndrome results in iron-deficient anemia and gastric hyperplasia [15].” The cited number 15 reference is wrongly listed as Grove et al 2010 and should instead be Iron-deficiency anaemia, gastric hyperplasia, and elevated gastrin levels due to potassium channel dysfunction in the Jervell and Lange-Nielsen Syndrome. Winbo A, Sandström O, Palmqvist R, Rydberg A. Cardiol Young. 2013 Jun;23(3):325-34.

The authors would like to apologise for any inconvenience caused.

